# Positive ageing perceptions among migrant Turkish and native Dutch older people: a matter of culture or resources?

**DOI:** 10.1186/s12877-017-0549-6

**Published:** 2017-07-21

**Authors:** Jane M. Cramm, Anna P. Nieboer

**Affiliations:** Department of Social Medical Sciences, Institute of Health Policy and Management, Erasmus University Rotterdam, P.O. Box 1738, Rotterdam, 3000 DR The Netherlands

**Keywords:** Aging perceptions, Resources, Culture, Turkish migrant, Apq-s

## Abstract

**Background:**

This study examined ethnic differences in ageing perceptions of migrant Turkish and native Dutch elders residing in Rotterdam, and explored whether such differences could be attributed to culture or resources (personal, physical, economic and/or social).

**Methods:**

This study was based on combined data from two research projects focusing on the health and well-being of community-dwelling elderly people in Rotterdam. The first dataset contained data from 994 native Dutch elders aged 70–99 years. The Rotterdam municipal register was used to randomly sample respondents, stratified by age group (70–74, 75–79, 80–84, and ≥85 years) and neighbourhood. Of the 2593 eligible respondents, 1075 returned filled-in questionnaires (41% response rate). Of these 1075 respondents a total of 994 were natives which is the sample we selected for the current study. The second dataset contained data from 680 Turkish migrants aged 65–90 years. All Turkish people aged ≥65 years were identified using the Rotterdam municipal register and invited to participate. In total, 680 Turkish respondents returned filled-in questionnaires (32% response rate; out of 2350).

Ageing perceptions were measured using the 21-item Ageing Perceptions Questionnaire–Short (APQ-S). Respondents were additionally asked about their current general health, income, education, marital status, age and gender.

**Results:**

The results of this study clearly reveal the importance of culture for all ageing perceptions among Turkish and Dutch elders. We found that age, health, and education were also important factors. For Turkish elders, health and education were the most important resources; for Dutch elders, age and health were most important in relation to ageing perceptions. Ageing perceptions were generally more negative among Turkish than among Dutch elders. Turkish elders reported more negative awareness of ageing, felt less in control of their ageing processes, and had more negative emotional reactions to ageing. They also believed more firmly that their ageing processes would have both negative and positive effects on their lives. Results revealed poorer health, lower income and educational levels among Turkish than among Dutch elders. In addition, many more Dutch than Turkish elders were single/widowed/divorced.

**Conclusion:**

Physical and personal resources, in addition to culture, are most important for positive ageing perceptions among native Dutch and migrant Turkish elders residing in Rotterdam, the Netherlands. These resources are health and education for Turkish elders, and health and age for Dutch elders. Overall, ethnicity remained a significant predictor of ageing perceptions when other resources were taken into account.

## Background

Ageing entails a complex process of adapting to a multitude of age-related physical, psychological, and social changes [1]. To effectively respond to this process, people create cognitive representations of ageing, i.e. ageing perceptions [2, 3]. These perceptions can be both positive and negative (e.g. [2]), and they can influence older people’s well-being [4, 5], health [2, 4, 6], quality of life [7, 8], loneliness [9], anxiety [10], and depressive symptoms [2, 10, 11].

Studies of ageing perceptions have investigated mainly individual differences therein in Western populations. We need an in-depth understanding of cross-cultural differences in ageing perceptions, given the evidence for such differences [12, 13]. Turkish migrants who arrived initially in the late 1960s and the 1970s currently form one of the largest groups of ethnic minorities in Western Europe. They show one of the strongest ageing trends and have been identified as one of the most vulnerable elderly groups [14]. Although older Turkish people in Europe represent an important study population, research investigating cultural differences in ageing perceptions between them and Western European natives is scarce.

### Cross-cultural differences in ageing perceptions

Previous research has suggested that ageing perceptions are not necessarily ethnically neutral [15]. A growing body of literature has provided evidence that these perceptions differ among cultures [16]. According to social representations theory [17, 18], views on ageing within a given culture are a form of shared cultural representation. They constitute systems of ideas, values, and customs related to ageing that are treated by members of the society as if they were established reality. As such, they are a mixture of accurate depictions of age-related changes and the views of older people in general [19], which are expected to differ between older Turkish people and natives in Western Europe [13]. For example, old age is considered to begin at a much earlier stage according to Turkish people than people from western European cultures [20, 21]. Furthermore, studies suggest that no consensus has been reached as to what constitutes being ‘old’, and that cultural differences may explain differences in this and other ageing perceptions. Furthermore, Turkish migrants are likely to have fewer resources compared with elderly native Europeans, which may cause difficulties in coping with old age. They might thus be at greater risk of developing maladaptive ageing perceptions, as supported by a few studies showing that elderly Turkish migrants felt old at a younger age [22] and had more adverse evaluations of their own ageing [23, 24]. The extent to which these differences in ageing perceptions can be attributed to differences in *culture* or *resources*, however, remains unclear.

### Resources

Perceptions of ageing play a vital role in older people’s ability to adjust adequately to loss and major life events (e.g. the loss or sudden severe illness of one’s spouse) [25]. Negative perceptions of ageing (i.e. having a severe loss frame) negatively influence adjustment to such life events because they limit the ability to see opportunities for substitution. Negative ageing perceptions, in turn, are affected by the availability of resources; more resources are associated with the ready availability of more opportunities, which are likely to weaken the loss frame. Research has shown that a stable and varied repertoire of such resources allows people to achieve instrumental goals, which are important for the achievement of overall well-being and compensation for losses that are associated inextricably with ageing [25, 26]. An important *physical resource* is health. The optimisation of opportunities for good health enables older people to take part in society actively and enjoy independent and high-quality lives [27]. Financial security has been identified as important *economic resource*, given that adapting to the process of ageing is generally easier when a person is without financial worries. Financial means help older people to feel less negative and worried, enabling them to cope better with ageing and life in general. Money, for example, helps not only to secure one’s basic physical needs (e.g. food, drink, warmth), but also to compensate for declines in physical health by enabling a person to acquire at-home formal assistance. Education is an important *personal resource*, as it provides intellectual skills to deal with the consequences of ageing and to maintain a positive frame of mind [28]. Researchers have underscored the importance of higher educational levels for the development of more favourable ageing perceptions [29, 30]. Gender-based differences in opportunities and living conditions are also known to accompany people across the lifespan. Older women in Europe, for example, are at greater risk of poverty than are older men [27]. Gender is evident as a *personal resource* in the context of life expectancy and cause of death. Although women tend to have longer life expectancies than men, they do not necessarily enjoy more years of better health or quality of life. The gender difference in life expectancy is thus smaller when years lived with disability are taken into account, implying that many women live longer with chronic disease or disability. Furthermore, older women in Europe report more psychological symptoms and greater use of medical consultation and treatment than do older men [27]. These differences may also result in different ageing perceptions. Actual age is another important *personal resource*; with increasing age, the ageing experience is increasingly framed in terms of physical decline or social loss and less in terms of continuous growth [28]. *Social resources*, such as having a spouse, children, and close friends, are also key in old age [1, 25]. Becoming old may not be perceived as alarming when a person can rely on others, for example, to help with groceries or provide a listening ear. In a study conducted among older Korean Americans, social resources were found to contribute to more positive views about ageing [31].

Depending on the availability of such resources, older persons are more or less likely to have positive (or negative) perceptions of ageing. Access to a variety of resources may reduce worries about the impact of ageing on one’s life and promote more positive ageing perceptions [25, 26, 28]. More specifically, those with a variety of resources more often think of old age as a time of development and less often associate ageing with physical decline and dealing with losses [28]. However, being a migrant or a member of an ethnic minority group can have a large impact on the accumulation of the aforementioned resources [32]. For example, long-term employment in low-paid manual-labour positions may lead to deficits in physical and economic resources, as one accumulates less financial means and may experience work-related physical discomfort in old age. Furthermore, being poorly educated may, for instance, impede understanding of the right to and procedures of elderly care [32]. On the other hand, migrant or ethnic minority elders may have more social resources than do native elders, due to strong ethnic bonds and cultural norms or values that promote interdependency and discourage divorce [32]. The greater availability of these social resources may offset some of the negative effects of the lack of other resources, thereby encouraging a more positive or less negative evaluation of ageing.

In summary, some evidence suggests that personal, physical, economic, and social resources, in addition to culture, can explain cross-cultural differences in ageing attitudes. However, findings are limited. As populations in nations across the world age [33], societal perceptions of older adults and people’s expectations about their own ageing processes are expected to influence the well-being of older adults, as well as policy decisions at the societal level. A better understanding of cross-cultural differences in ageing perceptions is a crucial step forward in navigating these challenges [13]. More research on the cultural meanings and significance of resources in relation to ageing perceptions in different populations is needed. Our lack of knowledge about cultural factors related to ageing may lead to the failure to plan appropriately for healthy ageing. We thus undertook the present research to compare ageing perceptions of natives and migrants in Rotterdam.

### Purpose of the study

This study quantitatively examined ethnic differences in ageing perceptions in a large sample of migrant Turkish and native Dutch elders residing in Rotterdam, and explored whether such differences could be attributed to culture or resources (personal, physical, economic and/or social). Based on previous findings, we hypothesised that ageing perceptions would be more negative among Turkish than among native elders (hypothesis 1). Furthermore, we expected that health (physical resource), education (personal resource), and income (economic resource) would be worse (hypothesis 2), but that social resources would be stronger (hypothesis 3), among Turkish than among Dutch elders.

## Methods

### Data collection

This study was based on combined data from two research projects focusing on the health and well-being of community-dwelling elderly people in Rotterdam, the Netherlands. Ethnic background was defined according to respondents’ countries of birth (i.e. Turkish for those born in Turkey, native Dutch for those born in the Netherlands).

The first dataset was gathered in February–April 2012 and contained data from 994 native Dutch elders aged 70–99 years [34]. The Rotterdam municipal register was used to randomly sample respondents, stratified by age group (70–74, 75–79, 80–84, and ≥85 years) and neighbourhood. The number of participants per neighbourhood was weighted according to the population ratio of the district. Only one person per address was allowed to participate. Participants were sent questionnaires with written invitations and self-addressed envelopes. Two reminders were sent in cases of non-response. Of 2890 people approached, 297 were excluded because they did not meet the study criteria [i.e. were not community dwelling (*n* = 67) or native Dutch (*n* = 205)] or were unable to participate due to serious medical problems (i.e. dementia) or death (*n* = 25). Of the 2593 eligible respondents, 1075 returned filled-in questionnaires (41% response rate). Of these 1075 respondents a total of 994 were natives which we used for the current study.

The second dataset contained data from 680 Turkish migrants aged 65–90 years, gathered in March–June 2015 and October 2015–February 2016. Data were not collected between June and October 2015 because many elderly Turkish individuals travel to Turkey during this period. All Turkish people aged ≥65 years were identified using the Rotterdam municipal register and invited to participate (census data). More than one person per address was allowed to participate. In total, 2350 respondents were sent questionnaires with written invitations and self-addressed envelopes. In the first data collection period (March–June 2015), the invitation and questionnaire were made available in Dutch and Turkish. As we noticed that the majority of respondents could not read Dutch, only the Turkish versions of the questionnaire and invitation were sent to respondents in the second data collection period (October 2015–February 2016). In cases of non-response, single reminders were sent, followed by at-home visits by ethnically matched interviewers. In total, 680 Turkish respondents returned filled-in questionnaires (32% response rate). Of the non-respondents, 213 were ineligible due to serious medical issues or death (*n* = 102), change of address (*n* = 110), or non-Turkish ethnic background (*n* = 1).

### Measures

#### Perceptions of ageing

Ageing perceptions were measured using the 21-item Ageing Perceptions Questionnaire–Short (APQ-S) [2, 4]. The APQ-S assesses ageing perceptions across seven (sub)dimensions, based on Leventhal’s self-regulation model [35]: timeline, or a person’s awareness of ageing, which can be constant (*chronic/acute*: e.g. “I always classify myself as old”) or vary over time (*cyclical*: e.g. “I go through phases of feeling old”); consequence, or the believed impact of ageing on various life domains, which can be positive (e.g. “As I get older, I get wiser”) or negative (e.g. “As I get older, I can take part in fewer activities”); perceived control over aspects related to ageing, which can be positive (e.g. “Whether I continue living my life to the full depends on me”) or negative (e.g. “Slowing down with age is not something I can control”); and emotional representations, comprised of negative emotional reactions toward one’s ageing (e.g. “I get depressed when I think about getting older”). Each dimension has three items, with responses ranging from 1 (‘totally disagree’) to 5 (‘totally agree’). The items of the *control negative* dimension were reverse coded, so that higher scores indicated more perceived control.

#### Physical resources

Respondents were asked to rate their perceived current general health on a five-point scale ranging from 1 (‘very bad’) to 5 (‘very good’). This measure is considered to be valid and robust; self-reported health has been proven to be a strong predictor of mortality, physical disability, and chronic illness [36–38]. Responses were dichotomised as indicating ‘poor’ (1, 2) and ‘good’ (3–5) health.

#### Personal resources

Respondents were asked to report the highest educational level completed in the Netherlands or abroad. For the Dutch sample, educational level was characterised using seven categories, ranging from ‘less than 6^th^ grade of elementary school’ to ‘higher vocational school and university’. For the Turkish sample, two additional categories were used: ‘no schooling’ and an option to write down other, unlisted, forms of schooling. This variable was dichotomised as completion of elementary school or less (1) or more than elementary school (0). The questionnaire also solicited information on age and gender.

#### Economic resources

Income level was determined based on respondents’ reported monthly household income, including social benefits, pensions, and alimony. Responses ranged from 1 (‘less than €1000 a month’) to 4 (‘€3050 a month or more’). ‘Do not know/do not want to tell’ was included as a fifth category. Income level was dichotomised, with ‘€1350 or more’ used as a reference category (below ‘€1350 in the Netherlands is considered to be below a minimum standard (Blommesteijn and Mallee 2009)).

#### Social resources

Respondents were asked to indicate whether they were married, divorced, widowed, single, or living together as one half of an unmarried couple. A dichotomous variable was created, with ‘divorced, single, or widowed’ serving as the reference category.

### Analyses

Sample characteristics were examined for the total study sample and separately for Dutch and Turkish respondents. To explore hypotheses 1–3, the significance of differences in ageing perceptions and resources between the Turkish and Dutch samples was tested using independent-samples *t* tests and chi-squared tests.

Bivariate associations were calculated to investigate the relationship between resources and ageing perceptions. Finally, stepwise regression analyses were performed for each dimension of ageing perception. Each regression analysis consisted of two steps. In the baseline model (model I), only ethnicity (culture) was regressed onto the ageing perception dimension. In the second model (model II), physical (health), personal (age, educational level, and gender), economic (income), and social (marital status) resources were entered into the equation. The results of regression analyses were compared between the Dutch and Turkish samples to investigate the relative contributions of the independent variables to all seven ageing perceptions.

## Results

### Sample characteristics

The Turkish sample was significantly younger than the Dutch sample [72.89 (5.03) vs. 78.66 (6.07) years; *p* < 0.001] and included fewer women (47.6% vs. 58.1%; *p* < 0.001; Table 1). Large differences were found between Turkish and Dutch older people in educational level (low, 80.9% vs. 15.5%; *p* < 0.001) and monthly household income (low, 84.7% vs. 34.2%; *p* < 0.001). Furthermore, more Turkish than Dutch older people reported being in poor health (72.6% vs. 44.5%; *p* < 0.001). Finally, fewer Turkish than Dutch elders were single, divorced, or widowed (29.1% vs. 57.4%; *p* < 0.001).

**Table 1:** Characteristics of the Turkish and Dutch samples

Variable (range)	Turkish (*n* = 680)		Dutch (*n* = 994)			
	M (SD)/%	Missing (%)	M (SD)/%	Missing (%)	*t* or *χ* ^2^	*p*
*Personal resources*						
Age (years)	72.89 (5.03)	0.0	78.66 (6.07)	0.0	20.843	<0.001
Education (low)	80.9	0.5	15.5	2.2	677.439	<0.001
Gender (female)	47.6	0.0	58.1	0.0	17.249	<0.001
*Physical resources*						
Health (poor)	72.6	0.6	44.5	1.9	122.359	<0.001
*Economic resources*						
Income (<€1350)	84.7	17.9	34.2	15.4	330.917	<0.001
*Social resources*						
Marital status (single/widowed/divorced)	29.1	0.5	57.4	1.5	123.829	<0.001

*M* mean, *SD* standard deviation

### Ethnic differences in ageing perceptions

As expected, significant differences in ageing perceptions were found between Turkish and Dutch respondents (Table 2). On average, Turkish respondents reported a more negative awareness of ageing, as shown by significantly higher mean chronic/acute and cyclical timeline scores. Furthermore, Turkish respondents felt significantly less in control of positive and negative aspects of the ageing process. Turkish elders also reported having significantly more negative emotional reactions to ageing, such as feeling down or depressed and angry. Finally, whereas Turkish elders believed more firmly that the ageing process would have negative impacts on their lives (e.g. ‘As I get older I can take part in fewer activities’ and ‘Getting older makes everything a lot harder’), they also experienced the positive consequences of ageing (e.g. ‘With age comes wisdom, personal growth, and feeling more appreciative of things’) more strongly than did native Dutch elders.

**Table 2:** Ageing perception scores in the Turkish and Dutch samples

	Turkish (*n* = 662^a^)	Dutch (*n* = 994)	*p*
*Timeline (negative awareness of ageing)*			
Chronic/acute	3.79 (0.90)	3.34 (0.91)	<0.001
Cyclical	3.55 (0.72)	2.85 (0.86)	<0.001
*Perceived consequences of ageing*			
Positive	3.60 (0.95)	3.40 (0.73)	<0.001
Negative	3.86 (0.91)	3.40 (0.81)	<0.001
*Feeling in control*			
Control over positive ageing effects	3.46 (0.97)	3.71 (0.68)	<0.001
Control over negative ageing effects	2.24 (0.83)	2.41 (0.74)	<0.001
*Negative emotional representations*	2.86 (1.02)	2.45 (0.84)	<0.001

^a^18 respondents did not fill in the ageing perceptions questionnaire leading to a total of 662 respondents

### Ageing perceptions: a matter of culture or resources?

As ethnic differences were found across the whole range of ageing perceptions, further analyses were performed to assess whether these differences are explained by culture or resources. Several significant but small associations were found between age and ageing perceptions (Table 3). For example, older respondents reported more chronic perceptions of their ageing and believed that their ageing had more negative and less positive consequences. A significant gender difference was found only for the negative control dimension; on average, male participants felt more in control of negative aspects related to ageing (e.g. becoming less mobile) compared with older women.

**Table 3:** Bivariate associations between ageing perception dimensions and resources

	Timeline chronic *(negative perceptions)*	Timeline cyclical *(negative perceptions)*	Consequence positive *(positive perceptions)*	Consequence negative *(negative perceptions)*	Control positive *(positive perceptions)*	Control negative *(positive perceptions)*	Emotional representations *(negative perceptions)*
*Cultural resources*							
Turkish ethnicity	0.235***	0.388***	0.120***	0.256***	−0.153***	−0.103***	0.212***
*Physical resources*							
Poor health	0.272***	0.373***	−0.02	0.427***	−0.182***	−0.206***	0.293***
*Personal resources*							
Age	0.100***	−0.043	−0.074**	0.104***	−0.018	−0.094***	−0.019
Low educational level	0.241***	0.276***	0.050*	0.235***	−0.150***	−0.128***	0.197***
Female gender	−0.026	−0.006	0.016	0.020	−0.043	−0.048*	0.031
*Economic resources*							
Low income	0.151***	0.260***	0.069**	0.199***	−0.059*	−0.108***	0.171***
*Social resources*							
Single/widowed/divorced	−0.013	−0.042	−0.039	−0.024	−0.007	−0.031	0.001

*p < 0.05, **p < 0.01, ***p < 0.001

Ethnicity was associated significantly with all ageing perceptions (Table 3). In general, Turkish ethnicity was associated positively with the negative ageing dimensions (chronic and cyclical timeline, negative consequences, and emotional representations) and negatively with the positive ageing dimensions (feeling in control over positive and negative ageing effects). The only exception, in line with the findings presented in Table 2, is the observed positive relationship between Turkish background and the positive consequences of ageing (wisdom, personal growth, and feeling more appreciative).

As expected, monthly household income and educational level were also associated significantly with all ageing perceptions. The directions of these associations were similar to those observed for Turkish ethnicity; low education and income levels were associated positively with the negative ageing dimensions and negatively with the positive dimensions, except for the positive consequences of ageing. Respondents with low incomes and educational levels were more aware and felt less in control of their ageing processes. Furthermore, they reported having more negative emotional responses toward their ageing. As with Turkish ethnicity, these respondents believed more strongly that ageing had negative impacts on several life domains, but also that ageing had positive effects. Poor health was associated with all ageing perceptions except the positive consequences of ageing. Respondents who were in poor health had more negative and less positive ageing perceptions compared with those in good health.

### Multivariate associations between resources and ageing perceptions

To examine whether ethnicity remained a significant predictor of ageing perceptions when the previously mentioned resources were taken into account, stepwise regression analyses were performed. Culture was related significantly to ageing perceptions in models I and II (Table 4). The direction and strength of these relationships were similar to those of the bivariate associations. Health, age, and education were also found to be important predictors of ageing perceptions. Although low income level was associated with all seven dimensions of ageing perceptions in the bivariate analysis, it showed no significant association in the adjusted analysis. Overall, low educational level was related to more negative and less positive ageing perceptions (e.g. stronger awareness of ageing and weaker belief in its positive influence).

**Table 4:** Unstandardized coefficients of the regression models for ageing perceptions

Variables	Timeline chronic/acute *(negative perceptions)*		Timeline cyclical *(negative perceptions)*		Consequence positive *(positive perceptions)*		Consequence negative *(negative perceptions)*		Control positive *(positive perceptions)*		Control negative *(positive perceptions)*		Emotional representations *(negative perceptions)*	
	Model I B (SE)	Model II B (SE)	Model I B (SE)	Model II B (SE)	Model I B (SE)	Model II B (SE)	Model I B (SE)	Model II B (SE)	Model I B (SE)	Model II B (SE)	Model I B (SE)	Model II B (SE)	Model I B (SE)	Model II B (SE)
*Cultural resources*														
Turkish ethnicity	.43 (.05)^***^	.36 (.08)^***^	.75 (.05)^***^	.69 (.07)^***^	.24 (.05)^***^	.33 (.07)^***^	.49 (.05)^***^	.40 (.07)^***^	−.22 (.05)***	−.22 (.07)^***^	−.20 (.04)***	−.17 (.07)**	.45 (.05)***	.29 (.08)***
*Physical resources*														
Poor health		.37 (.05)^***^		.47 (.05)^***^		−.12 (.05)^*^		.61 (.05)^***^		−.25 (.05)***		−.26 (.04)***		.42 (.05)***
*Personal resources*														
Age		.04 (.00)^***^		.02 (.00)^***^		−.01 (.00)		.03 (.00)^***^		−.01 (.00)***		−.02 (.00)***		.01 (.00)*
Low educational level		.24 (.06)^***^		.01 (.06)		−.13 (.06)^*^		.11 (.06)^*^		−.10 (.06)		−.12 (.06)*		.13 (.07)*
Female gender		−.07 (.05)^*^		−.02 (.05)		.08 (.05)		.02 (.05)		−.02 (.05)		−.01 (.05)		.02 (.05)
*Economic resources*														
Low income		.01 (.06)		.06 (.05)		.01 (.06)		.06 (.05)		.09 (.06)		−.02 (.05)		.04 (.06)
*Social resources*														
Single/widowed		.02 (.06)		.04 (.05)		.04 (.05)		−.04 (.05)		−.02 (.05)		−.02 (.05)		.04 (.05)
*R* ^2^	.05	.16	.17	.26	.02	.03	.07	.25	.02	.05	.02	.08	.06	.12

*p < 0.05, **p < 0.01, ***p < 0.001. *SE* standard error

The proportion of explained variance was remarkably low for most ageing perception dimensions. In the final model, this proportion was lower in the Turkish sample (Table 5) than in the Dutch sample (Table 6); it reached 25% only for the negative consequence dimension. In the Turkish sample, poor health and education were the most important resources for ageing perceptions. In the Dutch sample, poor health and age were the most important resources; education was not associated significantly with any ageing perception. For the timeline chronic dimension, women had more negative ageing perceptions than did men. While the explained variance among the Dutch is higher compared to the Turkish it is still relatively low, which indicated other resources may be more important when it comes to ageing perceptions among older people.

**Table 5:** Unstandardized coefficients (Bs) of the regression models for ageing perceptions in the Turkish sample

	Timeline chronic/acute *(negative perceptions)*	Timeline cyclical *(negative perceptions)*	Consequence positive *(positive perceptions)*	Consequence negative *(negative perceptions)*	Control positive *(positive perceptions)*	Control negative *(positive perceptions)*	Emotional representations *(negative perceptions)*
Variables	B(SE) CI	B (SE) CI	B (SE) CI	B (SE) CI	B (SE) CI	B (SE) CI	B (SE) CI
*Physical resources*							
Poor health	.22 (.09)^**^ .055 – .387	.35 (.07)^***^ .209 – .487	−.35 (.09)^***^ −.530 – −.163	.62 (.08)^***^ .458 – .785	−.38 (.10)*** −.565 – −.188	−.30 (.08)*** −.456 – −.133	.50 (.10)*** .306 – .696
*Personal resources*							
Age	.01 (.01) −.002 – .027	.00 (.01) −.010 – .014	−.01 (.01) −.030 – .003	.01 (.01) −.005 – .024	−.02 (.01)* −.034 – .000	−.01 (.01) −.022 – .007	.01 (.01) −.010 – .025
Low educational level	.38 (.10)^***^ .185 – .564	.10 (.08) −.058 – .259	−.16 (.11) −.369 – .050	.30 (.10)^**^ .110 – .482	−.25 (.11)* −.461 – .031	−.20 (.09)* −.381 – −.013	.17 (.11) −.051 – .394
Female gender	.12 (.08) −.048 – .284	.02 (.07) −.115 – .162	.09 (.12) −.096 – .270	.07 (.08) −.096 – .230	.05 (.10) −.138 – .238	.04 (.08) −.126 – .196	−.03 (.10) −.226 – .163
*Economic resources*							
Low income	.02 (.11) −.185 – .231	.01 (.09) −.168 – .180	.09 (.12) −.138 – .321	.01 (.10) −.199 – .210	.07 (.12) −.168 – .303	.16 (.10) −.042 – .362	−.12 (.12) −.359 – .129
*Social resources*							
Single/widowed	−.01 (.09) −.193 – .168	−.05 (.08) −.202 – .100	−.00 (.10) −.203 – .195	.03 (.09) −.143 – .212	−.01 (.09) −.313 – .096	−.02 (.05) −.184 – .166	.12 (.11) −.096 – .327
*R* ^2^	.06	.05	.00	.25	.03	.09	.07

*p < 0.05, **p < 0.01, ***p < 0.001. *SE* standard error, *CI* Confidence Interval

**Table 6:** Unstandardized coefficients (Bs) of the regression models for ageing perceptions in the Dutch sample

	Timeline chronic/acute *(negative perceptions)*	Timeline cyclical *(negative perceptions)*	Consequence positive *(positive perceptions)*	Consequence negative *(negative perceptions)*	Control positive *(positive perceptions)*	Control negative *(positive perceptions)*	Emotional representations *(negative perceptions)*
Variables	B(SE) CI	B (SE) CI	B (SE) CI	B (SE) CI	B (SE) CI	B (SE) CI	B (SE) CI
*Physical resources*							
Poor health	.43 (.06)^***^ −.896 – .678	.54 (.06)^***^ .424 – .649	.01 (.05) −.093 – .107	.60 (.05)^***^ .497 – .699	−.18 (.05)*** −.273 – −.086	−.26 (.05)*** −.353 – −.155	.38 (.06)*** .271 – .496
*Personal resources*							
Age	.05 (.01)^***^ .308 – .542	.03 (.01)^***^ .016 – .035	−.00 (.00) −.012 – .005	.05 (.00)^***^ .037 – .054	−.01 (.00)*** −.021 – −.005	−.03 (.00)*** −.035 – −.019	.01 (.01)* .000 – .019
Low educational level	.09 (.08) .037 – .057	−.05 (.08) −.205 – .106	−.09 (.07) −.227 – .050	−.05 (.07) −.185 – .094	.03 (.07) −.103 – .155	−.08 (.07) −.214 – .060	.10 (.08) −.053 – .258
Female gender	.12 (.08)^***^ −.422 – −.166	−.04 (.06) −.165 – .080	.10 (.06) −.007 – .212	−.05 (.06) −.157 – .064	−.03 (.05) −.129 – .076	−.03 (.06) −.141 – .076	.04 (.06) −.083 – .163
*Economic resources*							
Low income	.03 (.07) −.144 – .164	.07 (.07) −.068 – .199	−.04 (.06) −.163 – .075	.11 (.06) −.007 – .233	.07 (.06) −.043 – .180	−.09 (.06) −.211 – .024	.12 (.07) −.014 – .253
*Social resources*							
Single/widowed	.03 (.07) −.109 – .175	.08 (.07) −.056 – .215	.07 (.06) −.056 – .185	−.09 (.06) −.211 – .033	.03 (.06) −.079 – .147	.04 (.06) −.116 – .124	−.02 (.07) −.159 – .113
*R* ^2^	.17	.14	.00	.25	.03	.09	.07

*p < 0.05, **p < 0.01, ***p < 0.001. *SE* standard error, *CI* Confidence Interval

## Discussion

Although ethnic differences in ageing perceptions may be rooted in socio-economic and health differences, cultural differences may also shape the ageing experience [22–24]. Furthermore, research clearly emphasized the benefits of considering multiple predictors simultaneously when it comes to investigating ageing perceptions [39]. The results of this study clearly reveal the importance of culture for all ageing perceptions among Turkish and Dutch elders residing in Rotterdam. This is in agreement with earlier studies [22–24, 39]. We additionally found that age, health, and education were also important factors. For Turkish elders, health and education were the most important resources; for Dutch elders, age and health were most important in relation to ageing perceptions. The explained variance in the final model was low overall and was greater in the Dutch than in the Turkish sample, indicating that factors not included in the model explain ageing perceptions among Turkish elders. Thus, additional cultural factors, such as acculturation [40] and religion [41], should be taken into account in future studies.

Ageing perceptions were generally more negative among Turkish than among Dutch elders, supporting hypothesis 1. Turkish elders indeed reported more negative awareness of ageing, felt less in control of their ageing processes, and had more negative emotional reactions to ageing. They, however, believed more firmly that their ageing processes would have both negative and positive effects on their lives. Culture seems to lead to certain expectations regarding growing older which also include personal growth and feeling more appreciative of life. Regarding all the other six ageing perceptions our hypothesis was confirmed; overall a Turkish background leads to more negative ageing perceptions and less positive ones compared to native Dutch elderly.

The study results also support hypotheses 2 and 3. They clearly revealed poorer health and lower income and educational levels among Turkish than among Dutch elders (hypothesis 2), in agreement with previous findings [32]. In addition, many more Dutch than Turkish elders were single/widowed/divorced (hypothesis 3).

Although the response rate was lower for Turkish than for Dutch elders in this study, it was comparable to those in other surveys conducted in this population [42]. We made a minimum of two contact attempts for the Turkish sample. To improve the response rate, this number should be increased to six contact attempts, which was not feasible in our study [22, 42]. Another limitation of this study is its cross-sectional design, which hampered the ability to draw causal conclusions. Thirdly, this study was conducted only among Turkish and Dutch elders. It would be interesting to conduct a similar study among, for example, Moroccan elders in Rotterdam, as they are the second largest migrant group in the city. Finally, we merged two datasets, which limited our choice of variables representing the resources examined. Future research should use a more extensive set of variables to capture a wider repertoire of resources.

## Conclusion

We can conclude that physical and personal resources, in addition to culture, are most important for positive ageing perceptions among native Dutch and migrant Turkish elders residing in Rotterdam, the Netherlands. These resources are health and education for Turkish elders, and health and age for Dutch elders. Overall, ethnicity remained a significant predictor of ageing perceptions when other resources were taken into account. However, a large part of the variation in ageing perceptions remained unaccounted for, especially among Turkish elders. Thus, we have solved only a small part of this puzzle. Research investigating additional cultural aspects, such as acculturation and religion, is needed. Such efforts may increase our understanding of ethnic differences in ageing perceptions.
